# Meningeal lymphatics and their role in CNS disorder treatment: moving past misconceptions

**DOI:** 10.3389/fnins.2023.1184049

**Published:** 2023-07-12

**Authors:** Alexandra Melloni, Longsha Liu, Vivek Kashinath, Reza Abdi, Khalid Shah

**Affiliations:** ^1^Center for Stem Cell and Translational Immunotherapy, Harvard Medical School, Boston, MA, United States; ^2^Department of Neurosurgery, Brigham and Women's Hospital, Harvard Medical School, Boston, MA, United States; ^3^Department of Nephrology, Brigham and Women's Hospital, Harvard Medical School, Boston, MA, United States; ^4^Harvard Stem Cell Institute, Harvard University, Cambridge, MA, United States

**Keywords:** central nervous system, lymphatic drainage, immunotherapeutics, meningeal lymphatics, brain tumors, neurodegenerative disease

## Abstract

The central nervous system (CNS) was previously thought to lack lymphatics and shielded from the free diffusion of molecular and cellular components by the blood–brain barrier (BBB) and the blood–cerebrospinal fluid barrier (BCB). However, recent findings have redefined the roles played by meningeal lymphatic vessels in the recruitment and drainage of lymphocytes from the periphery into the brain and the potentiation of an immune response. Emerging knowledge surrounding the importance of meningeal lymphatics has the potential to transform the treatment of CNS disorders. This review details the most recent understanding of the CNS-lymphatic network and its immunologic implications in both the healthy and diseased brain. Moreover, the review provides in-depth coverage of several exciting avenues for future therapeutic treatments that involve the meningeal lymphatic system. These therapeutic avenues will have potential implications in many treatment paradigms in the coming years.

## Introduction

The central nervous system (CNS) has traditionally been described as an immune-privileged organ due to the many physical barriers that protect the brain from insults, expression of immunoregulatory molecules, and low, if not absent, presence of traditional lymphatics and major histocompatibility complex class II-expressing antigen-presenting cells (APCs) at a steady state (Medawar, 1948). These factors, combined with the Medawar discovery of immune tolerance wherein normally rejected allografts introduced into the CNS resulted in secondary systemic antigen-specific tolerance, led to the prevailing belief that immune surveillance in the CNS is significantly limited and lacks the necessary connection to induce a sufficient immune response to CNS antigens (Billingham et al., 1953). However, in recent years, with the advent of new tracer technologies, a catalytic shift in the understanding of immune privilege and fluid dynamics as it pertains to the CNS has begun. The presence of a connection between the CNS and cervical lymph nodes was discovered, and studies have demonstrated the mounting of a potent immune response via the lymph nodes when antigens are delivered to the CNS (Harling-Berg et al., 1989). This (re)discovery of CNS immune surveillance through CNS fluid drainage via the lymphatic system and the crucial role played by meningeal lymphatics—a key communicator between the CNS and peripheral immune system—necessitates a revisitation on CNS lymphatic drainage and its implication in the development and treatment of CNS disorders.

## Exploring lymphatics: from a CNS perspective

**Figure 1:**
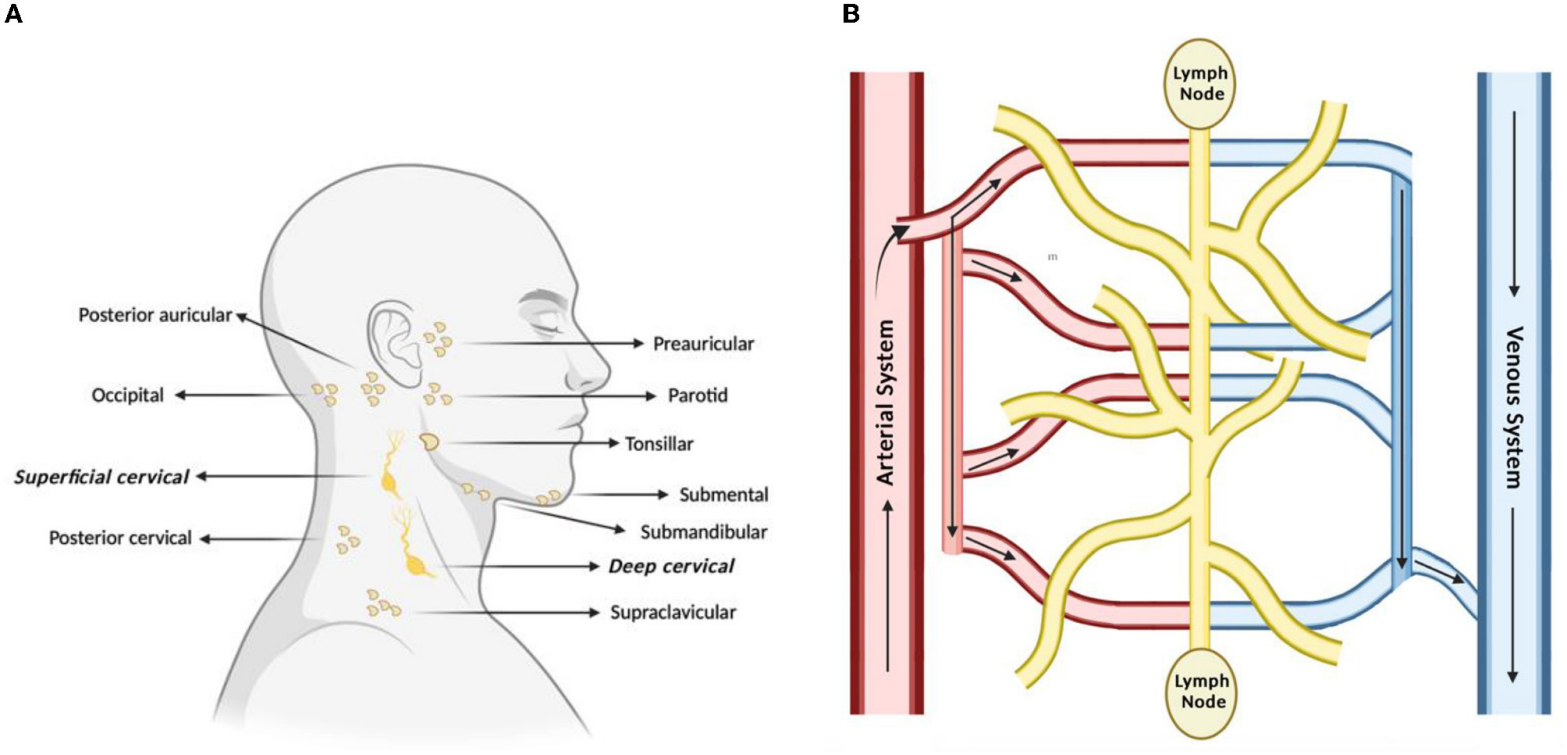
Lymph nodes play a vital role in lymphatic drainage and immune surveillance. **(A)** Of the many subsets of lymph nodes in the head and neck, the immune surveillance process occurs in the superficial and deep cervical lymph nodes. **(B)** Process of lymphatic drainage begins with fluid extravasation from blood vessels, which filter through the lymph nodes, before entering back into blood circulation.

The lymphatic system is classically understood to be involved in maintaining general tissue fluid balance, lipid absorption in the intestines, and immune surveillance. It is a unidirectional transport system that collects a variety of waste fluids, cells, and macromolecules that have extravasated from blood vessels and returns them to venous circulation (Hampton and Chtanova, 2019). The multi-step lymphatic process begins with the drainage of interstitial fluid (ISF) in the blind-ended capillaries of body tissues and organs to the lymphatic vessels via ISF pressure gradients induced by muscle contraction and arterial pulsations. Subsequently, the collected lymph that is now enriched with potential peptides for antigen recognition is transported to lymph nodes and presented to the lymphocytes by resident dendritic cells. Lymph then re-enters and is incorporated back into blood circulation via the thoracic duct (Randolph et al., 2017). This immune surveillance process occurs in the brain's superficial and deep cervical lymph nodes (Louveau et al., 2015), which is shown in relation to the rest of the lymphatic system in Figure 1. Overall, the importance of the lymphatic system in immune surveillance cannot be underemphasized, as the presentation of fluids and associated antigens via lymphatic vessels enables the mounting of immune responses at the LN and, conversely, lymphatic drainage of antigens permits clearance of immune cells from sites of inflammation (Alitalo, 2011) (reference Table 1 for more details).

**Table 1:** Components of the lymphatic system.

**Component**	**Structure**	**Function**
Lymph	• Watery fluid that flows through the lymphatic system	• Passing from blood to the body tissues, waste products are collected • Lymph then drains to the lymphatic vessels and is transported to the lymph nodes, where harmful products are filtered out, and lymphocytes within the nodes help to fight infection
Lymphatic vessels	• Composed of lymphatic endothelial cells (LECs) • Highly concentrated in the skin and mucous membranes • Contains blood plasma, extra fluid, macromolecules, dietary lipids	• Helps to reabsorb fluid and important antigens and antigen presenting cells (APCs) into lymph nodes • Exports immune cells, important for migration and potentiation of immune surveillance response
Lymphoid organs	• Location of lymphocyte production/secretion (composed of primary and secondary lymphoid organs)	• **Primary:** the location of immature lymphocyte development (thymus and bone marrow) • **Secondary:** location of antigen localization, begins adaptive immune response (spleen, lymph nodes, adenoids, tonsils, and Peyer's patches)
Lymphocytes	• Immune cells of the lymphatic system; T cells, B cells, NK cells	• CD8+ T cells kill infected cells, CD4+ helper T cells secrete cytokines • B cells produce antibodies to target antigens • NK cells control infections by secreting IFNγ and TNFα
Lymph nodes (LNs)	• A collection of lymphatic tissue • Secondary lymphoid organs • House and contain T cells, B cells, plasma cells, macrophages	• Responsible for cleansing and monitoring the lymph as it is filtered through the system • Location of immune response potentiation

### Glymphatic system

The glymphatic system is a recently discovered macroscopic waste drainage system responsible for the interconnection between cerebrospinal fluid (CSF) and ISF as they mix while passing through the glia limitans (Engelhardt et al., 2017) and is comprised of vessels that form pathways that traverse the brain through paravascular spaces via aquaporin-4 channels (Desland and Hormigo, 2020). The system, which bears remarkable similarities in function to the lymphatic system, was first characterized *in vivo* in 2012 by observing the path of CSF fluorescent tracers as they traversed and exited the CNS parenchyma (Iliff et al., 2012). Notably, while the glymphatic system constitutes a major debris clearance pathway, it is suggested that the glymphatic system may also provide a route for the spread of tumor cells or other aberrated cells associated with CNS disorders; therefore, it is an important candidate of study in neurological disorders (Tamura et al., 2020). At present, however, the role and mechanism by which the glymphatic system contributes to antigen drainage from the brain parenchyma remains contentious and questioned (Hladky and Barrand, 2022).

### Meningeal lymphatics

The definite discovery of functional lymphatic vessels in the brain, located next to highly vascular meninges, has represented a paradigm shift in the understanding of CNS fluid balance, immune surveillance, and immune response. Catalyzed by the recent publications of two key studies, the newly discovered meningeal lymphatics are located in the dorsal and basal positions beneath the skull, with the basal meningeal lymphatic vessels being anatomically different from their dorsal counterparts and more effective at draining fluid from the CNS (Ahn et al., 2019; Hu et al., 2020). Importantly, both lymphatic vessels were noted to contain T-cells and MHC II-expressing APCs that drain from the subarachnoid space into the deep cervical lymph nodes (Desland and Hormigo, 2020). More recent studies have provided gradually increasing findings that implicate meningeal lymphatic vessels in the drainage of CSF and lymph into the peripheral blood volume to act in immune surveillance (Tamura et al., 2020); however, the process of lymphangiogenesis, as it occurs in the meningeal lymphatic vessels, is yet to be fully elucidated. Most of what is known about their functions can be illuminated by the structure; there are significant architectural differences between the meningeal and peripheral lymphatic vessels. Meningeal lymphatic vessels cover a smaller tissue area, contain fewer branches, and have a smaller diameter than their peripheral counterparts (Raper et al., 2016). These characteristics may suggest that their surrounding environment may not permit increased branching and vessel growth when needed and therefore could pose a problem for treatment as some conditions that involve aberrations in lymphatic drainage are treated with agents, such as VEGF-C (vascular endothelial growth factor C), to increase lymphangiogenesis. Nonetheless, the increase in MLVs could potentially reduce pressure on the brain and have implications for treatments for brain injury. Overall, the discovery of MLVs unveils an important connection between the CNS and peripheral immunity that was formerly unknown—one that has since modified the understanding of the CNS and its ability to potentiate an immune response.

## Migration of immune cells

The CNS was formerly thought to lack a dynamic innate immune response (Engelhardt et al., 2017). However, the ability of immune cells to migrate to the CNS via meningeal lymphatic drainage has shed light on how these cells can track areas of inflammation. Following an inflammatory reaction in the brain, lymphatic endothelial cells that comprise lymphatic vessels produce chemokines that attract immune cells, such as dendritic cells (DCs) and neutrophils, from the periphery to the lymph nodes, where they can then potentiate an immune response (Papadopoulos et al., 2020). Thus, these immune cells can enter the brain retrograde via afferent lymphatics that connect to draining lymph nodes, where they otherwise could not normally migrate into the brain through other methods.

### Dendritic cells

In the case of DCs, CCL19, and CCL21 are two important chemokines required for dendritic cell trafficking to lymph nodes and increased lymph node expansion (Ousman and Kubes, 2012), respectively. Both CCL19 and CCL21 are ligands of the chemokine receptor CCR7. It is important to note that the migration of DCs to areas of inflammation are cytokines IL-1β, TNF-α, and prostaglandin-E2, which increase CCR7 expression (Papadopoulos et al., 2020). Conversely, immunosuppressive agents, such as IL-10, TGF-β, and Resolvin E1, can inhibit DC migration and thereby decrease their recruitment to the brain and other systems.

### Neutrophils

Compared to dendritic cells, the chemokine receptor CCR7 is less important in neutrophils. However, the chemokine receptor CXCR4 does play a role in neutrophil migration from bone marrow to areas of inflammation, but it is not required for neutrophil entry into the lymphatic system (Hampton and Chtanova, 2019). Importantly, the cell surface receptor CD11b is emerging as an important regulator of neutrophil migration in the lymphatic system, as are inflammatory cytokines such as TNF-α (Hampton and Chtanova, 2019).

### Adaptive immune cells

T and B cells, part of the adaptive immune response, are not generally present in the CNS parenchyma of healthy brains (Swartz et al., 2008; Engelhardt et al., 2017) but can be found in the CSF, pointing to the use of the meningeal lymphatic system for the trafficking of these cells. CD4^+^ T cells, such as dendritic cells, also use CCL19 gradients to migrate to lymph nodes in both a healthy and inflamed brain environment. One possible route of entry of T cells (in their activated state) into the brain is via migration from blood vessels into the stroma of the choroid plexus (Goverman, 2009; Rua and McGavern, 2018). From there, T cells may cross the blood-CSF barrier into the subarachnoid space. B cells, by contrast, are both formed and matured in the bone marrow. Their main role in adaptive immunity is to produce antibodies to target antigens. They use different afferent lymphatics to migrate to deep cervical lymph nodes than T cells, and their migration to areas of inflammation may also require CCR7 (Harling-Berg et al., 1989; Eibel et al., 2014) as well as IL-4 and IL-2. Importantly, adaptive immune cells have the potential to play a role in lymphangiogenesis as activated B cells have been shown to produce VEGF-A in times of inflammation (Lucas and Tamburini, 2019). This product has been shown to cause an increase in lymph node size and lymphangiogenesis (Lucas and Tamburini, 2019), which could possibly be a potential treatment option for conditions in which lymphangiogenesis is negatively affected. T cells have also been shown to play a role by helping regulate the expansion of LECs (Lucas and Tamburini, 2019). However, meningeal lymphatics can still undergo lymphangiogenesis, but this process has not been outlined well in a neuroinflammation environment (Louveau et al., 2016).

## Meningeal lymphatics and neurological disorders

Recent *in vivo* studies have discovered a correlation between CSF lymphatic outflow and age, with the outflow reduced significantly in older mice compared with younger mice (Ding et al., 2021). Thus, it is important to consider how the dysfunction of meningeal lymphatic vessels, either via altered or impaired vessel outflow, can lead to a host of age-related neurodegenerative and neuroinflammatory diseases (Weller et al., 2010; Da Mesquita et al., 2018; Sun et al., 2018).

### Alzheimer's disease

A neuropathological hallmark of Alzheimer's disease is amyloid beta (Aβ) peptide accumulation and its subsequent plaque deposition in the brain. While the exact dynamics between Aβ accumulation and the meninges remain unclear, it is worth to note that one of the first isolations of Aβ protein was in the meningeal tissue homogenates of Alzheimer's patients (Joachim et al., 1988). From recent studies, however, it can be reasonably stated that meningeal lymphatic vessels are at least partially responsible for the dysfunctional clearance of Aβ from the brain. For one, transgenic mice that are absent of meningeal lymphatics face increased delays in macromolecule elimination from the brain (Aspelund et al., 2015). Moreover, in another study, the disruption of meningeal lymphatic vessels resulted in the exacerbation of Aβ pathology, with increased deposition of Aβ being found in the meninges, increased Aβ plaque load found in the hippocampus (Mentis et al., 2021), and elevated levels of macrophages detected that were recruited to the Aβ aggregates. The involvement of the glymphatic pathway, which also has been identified to play a role in Aβ paravascular clearance (Iliff et al., 2012; Peng et al., 2016) cannot be underlooked either. Indeed, a study investigating mice with deficiencies in both meningeal lymphatic vessels and glymphatic clearance observed a reduction in cognitive function compared to the control, with increased microglial expression and elevated hippocampal neural apoptosis (Mentis et al., 2021). Taken together, these studies provide compelling evidence of lymphatic drainage dysfunction—through both meningeal lymphatic vessels and the glymphatic pathway—in contributing to Alzheimer's pathology. Moving forward, it will be crucial to better characterize the exact mechanism of meningeal lymphatic vessels in response to elevated levels of Aβ.

### Parkinson's disease

Parkinson's disease involves an accumulation of the protein α-synuclein in the brain, where similar to Alzheimer's disease, the clearance of aggregated proteins from affected areas is essential for treatment. While the role meningeal lymphatic vessels play in α-synuclein still remains to be better elucidated, a study performed by Zou et al. in 2019 showed that the blockade of the meningeal lymphatic drainage in mice expressing the A53T point mutation of the human α-synuclein protein via ligation of deep cervical lymph nodes led to an increase in symptoms that are typical hallmarks of Parkinson's disease (Zou et al., 2019). This finding supported the theory that aberrations in lymphatic drainage to lymph nodes could be a major pathogenic feature of neurodegenerative diseases, such as Parkinson's disease, and this could have implications for treatments of such disorders. Recently, a study of patients with idiopathic Parkinson's disease provided even more conclusive evidence of the link between meningeal lymphatic vessels and Parkinson's pathology as idiopathic Parkinson's patients exhibited significantly lower meningeal lymphatic vessel outflow and reduced perfusions in the deep cervical lymph nodes (Ding et al., 2021). Moreover, taken *in vivo*, the idiopathic Parkinsonian models of transgenic mice with blocked meningeal lymphatic vessels had increased α-synuclein pathology and worsened motor and memory deficits. Furthermore, to confirm that the reduced flow rate through the meningeal lymphatic vessels was contributed primarily by impaired meningeal lymphatic uptake, a follow-up experiment from the same study injected gadobutrol intravenously in mice to prevent macromolecules from passing through the glymphatic system. Finally, the study suggested that impaired meningeal lymphatic drainage in idiopathic Parkinson's disease may have been caused by macrophage-induced meningeal inflammation in response to increased α-synuclein levels. Overall, from both Alzheimer's and Parkinson's studies, it is becoming evident that meningeal lymphatic vessel dysfunction is involved in protein-aggregation-related neurological disease pathology.

### Multiple sclerosis

Multiple sclerosis (MS) is caused by the targeting of the myelin sheath by the immune system, leading to cognitive and motor deficits. The complete etiology of this disorder in the immune system is unknown and the role that meningeal lymphatics plays in MS also remains highly speculative. However, there exists an inextricable link between lymphatic drainage and the migration of immune cells that are heavily involved in MS pathophysiology. In particular, the overactivation of auto-reactive T-cells and the inflammation of meningeal B cells are thought to be major contributors to MS cortical pathology (Sabatino et al., 2019). Recently, a study using the common animal model of induced experimental autoimmune encephalitis (EAE) provided more compelling evidence, noting that meningeal lymphatic ablation resulted in diminished disease pathology and reduced inflammatory response from auto-reactive T cells (Louveau et al., 2018). Critically, this finding suggests a key role of meningeal lymphatics in regulating the inflammatory process and necessitates a deeper investigation of meningeal lymphatic vessels as a therapeutic target.

### Traumatic brain injury

Affecting millions of people globally every year, TBI is of great interest due to the increasing evidence of its involvement in increasing the risk and exacerbating the pathology of numerous other neurological disorders later in life (Dams-O'Connor et al., 2016). Relevantly, studies have recently shown that TBI, even in its mild forms, can result in meningeal lymphatic dysfunction. In a relatively detailed study compared to the otherwise paucity in the characterization of meningeal lymphatic vessel dysfunction and other neurological disorders, Bolte et al. in 2020 observed a significant decrease in drainage of beads to the deep cervical lymph nodes 2 h following a TBI, with the meningeal lymphatic drain dysfunction persisting for 1-month post-injury in TBI mice via fluorescent tracers. Moreover, the study noted in parallel substantial lymphatic vasculature morphological changes within 1–2 weeks following injury; interestingly, however, the observed lymphangiogenesis following mild TBI reverses back to pre-injury characteristics within a month, which may help explain the restoration of proper meningeal lymphatic drainage. Furthermore, pre-existing deficits in meningeal lymphatic function before injury showed worsened cognitive outcomes afterward, with mice that underwent meningeal lymphatic photoablation prior to TBI scoring lower in memory performance tests such as the novel location recognition test. Overall, the study potently demonstrated the influence of TBI on meningeal lymphatic dysfunction and showed a causal link between TBI and exacerbated cognitive dysfunction.

### Brain tumors

In addition to affecting the brain environment in various types of neurological disorders, the meningeal lymphatic drainage system can also affect the growth of many kinds of brain tumors. Notably, metastasis of tumor cells from the primary tumor in the CNS to other organs is one of the most accurate and important predictors of patient survival from the disease (Shayan et al., 2006; Podgrabinska and Skobe, 2014). Lymphatic vessels can provide a nurturing environment for tumor growth, and melanoma patients with metastasis to the lymph nodes have been found to be more likely to experience disease recurrence despite complete resection of the primary tumor (Alitalo, 2011; Padera et al., 2016). It is thus theorized that the lymphatic system is directly implicated in leading to local metastasis through tumor-draining lymphatic vessels that could host tumor cells and cause their spread to other areas in the lymphatic system (Venero Galanternik et al., 2016). Moreover, it has been demonstrated with animal models that peripheral T-cells can be stimulated in response to CSF drainage of soluble tumor antigens to the deep cervical lymph nodes (Hu et al., 2020) and that lymphangiogenesis increases the probability for tumor cells to enter into the lymphatic vessels (Swann and Smyth, 2007; Lund et al., 2016). Conversely, inhibiting the metastasis to the lymph nodes decreases metastasis to other areas, further supporting the theory that the lymph nodes are key areas of tumor cell spread. More recently, an *in vivo* study provided even more compelling supporting data, citing extensive lymphatic phenotypic and genotypic remodeling of the dorsal meningeal lymphatic vessels in glioma and metastatic melanoma mice models (Hu et al., 2020). Interestingly, the disruption of just the dorsal meningeal lymphatic vessels resulted in impaired intratumor fluid drainage, reduced efficacy of anti-PD-1/CTLA-4 immune checkpoint blockade combination therapy, and decreased tumor cell migration to the deep cervical lymph nodes.

It is therefore important to note that manipulation of the meningeal lymphatic system has the potential to either abate tumors or provide an environment for tumor growth. While increased lymphatic drainage due to lymphangiogenesis may increase the drainage of soluble tumor antigens to provoke an anti-tumor immune response, it may also increase the potential for tumor spread through the lymphatics (Lund et al., 2016). Nonetheless, the studies to date have highlighted the importance of meningeal lymphatic vessels in tumor development and immune response; moving forward, it is essential to consider how we can efficiently modulate meningeal lymphatics in promoting an anti-tumor environment.

## Meningeal lymphatic manipulation: implications for CNS therapy

Recent discoveries regarding the meningeal lymphatic drainage system present implications for the treatment of a variety of CNS disorders via the manipulation of the meningeal lymphatic drainage system. Currently, only one major treatment for meningeal lymphatic drainage has been well characterized, which is the virally mediated or mRNA-mediated delivery of vascular endothelial growth factor C (VEGF-C), a central molecule in driving lymphatic drainage. Treatments with VEGF-C have been previously shown to substantially increase meningeal lymphatic vessel diameter and boost peripheral lymphangiogenesis (Da Mesquita et al., 2018) without any off-target effects detected. Perhaps even more importantly, 1-month post-treatment, aged mice were observed to have significantly elevated rates of CSF tracer influx from the deep cervical lymph nodes to the brain parenchyma (Da Mesquita et al., 2018).

Notably, the method of delivering exogenous VEGF-C has been exemplified well through the research of Song et al., in which delivery of VEGF-C led to increased immune cell recruitment and a more potent immune response in brain tumor mice models (Song et al., 2020). The exogenous expression of VEGF-C promoted quick clearance of the tumor and a longer-lasting anti-tumor memory response (Song et al., 2020). Additionally, the research performed by Penco-Campillo et al. is concordant with that of Song et al. In their study of medulloblastoma, they revealed that all the principal tumor subgroups (Wingless, Sonic Hedgehog, Group 3, Group 4) experienced a decrease in tumor growth by VEGF-C (Penco-Campillo et al., 2020) as increased lymphangiogenesis led to a subsequent increase in lymphatic drainage. These findings are further corroborated by studies pointing to increased activation of anti-tumor immunity in the lymph nodes following increased lymphatic drainage from lymphangiogenesis; tumor lymphangiogenesis in melanoma patients, for example, increased T-cell infiltration—especially CD8 T-lymphocytes—to the lymph nodes (Fankhauser et al., 2017). Moreover, in a separate study conducted on aged TBI mice, delivery of VEGF-C decreased levels of allograft inflammatory factor 1 (Iba1), a well-known marker for microglia, in the contralateral hemisphere of the injury site and reversed Iba1 immunoreactivity levels back to those more akin to young TBI mice (Bolte et al., 2020). The findings suggest that boosting meningeal lymphatic drainage may diminish gliosis and ultimately reduce incidence rates of subsequent neurological disorders following TBI.

Overall, the augmentation of lymphatic drainage in the meninges, particularly in aged mice, maybe a potent therapeutic strategy to treat numerous neurological diseases. For neurodegenerative diseases, such as Alzheimer's and Parkinson's disease, where aberrant lymphatic drainage is a factor in disease pathology, novel treatments that enhance clearance of CSF/ISF macromolecules and help clear debris, such as Aβ and α-synuclein, respectively, could be particularly beneficial (Xin et al., 2018). Furthermore, for neuroinflammatory diseases, such as MS, enhancing lymphatic drainage from the deep cervical lymph nodes may lower the presence of antigens in the cervical lymph nodes that thereby weakens any potentially harmful autoimmune response (Hsu et al., 2019). Finally, it is important to note that lymphatic vessels are key in the discussion of tumor immunotherapy since they can transport antigens by dendritic cells to lymph nodes, a process that is relevant for the activation of the tumor-specific T-cell response and leads to the potentiation of an immune response (Garnier et al., 2019). In fact, in a recent study, the overexpression of VEGF-C was found to strengthen the anti-tumor effectiveness of anti-PD-1 immunotherapy (Song et al., 2020), indicating that a bimodal form of treatment could be the most effective at treating certain subsets of tumors. Thus, moving forward, it will be important to further characterize VEGF-C therapy in treating numerous neurological symptoms and especially explore synergistic strategies that leverage meningeal lymphatic drainage augmentations.

## Meningeal lymphatics in clinical trials

There are already several ongoing clinical trials that are translating preclinical findings about meningeal lymphatics and neurological disorders to early-stage clinical settings. While the full library can be found on ClinicalTrials.gov, there are several that are particularly relevant. A summary of these particularly relevant clinical trials can be found in Table 2. “Lymphatic system health in Alzheimer's disease” (ID: NCT04205539) was a phase I study seeking to characterize differences in lymphatic health among Alzheimer's disease patients. In the study design, the researchers are using dexmedetomidine, a short-acting agent, to induce a sleep-like state to assess interstitial fluid convection and free water diffusion in patients. The latter analysis method is particularly interesting and maybe a promising way to test the ability of the lymphatic system to remove extracellular debris. Similarly, “Quantitative imaging of brain glymphatic function in humans” (ID: NCT04768101) is a Phase I study aiming to use a novel magnetic resonance imaging method that is sensitive to lymphatic drainage dysfunction to assess the relationship between Alzheimer's disease-related dementias and CNS lymphatic function in patients. “Effects of exercise on glymphatic functioning and neurobehavioral correlates in Parkinson's disease (FIGHTPD)” (ID: NCT04140708) is another interesting ongoing study attempting to test whether exercise potentiates glymphatic function and would therefore increase amyloid beta clearance from the brain and improve neurobehavioral outcomes. Additionally, “The role of meningeal lymphatic vessels in the absorption of chronic subdural hematoma and its injury mechanism” (ID: NCT05426889) is a study currently recruiting participants aimed at discovering and optimizing the role that lymphatics play in the absorption of these hematomas and use the knowledge to develop targeted treatment down the line. Finally, “Meningeal inflammation on 7T MRI as a tool for measuring and predicting ocrelizumab response in multiple sclerosis” is a Phase I trial seeking to use 7 tesla MRI to identify meningeal inflammation as a predictor for the effectiveness of the drug ocrelizumab for the treatment of multiple sclerosis. It is speculated that meningeal inflammation, caused by circulating B-cells, may be the cause of gray matter damage to the brain; consequently, the use of ocrelizumab, which destroys B-cells, may reduce meningeal inflammation levels in patients. Overall, there is an enormous potential for meningeal lymphatics in therapy, and the clinical trials ongoing today are paving the way to better prepare for future translations from the bench to the bedside.

**Table 2:** Meningeal lymphatics in clinical trials.

**Study**	**Aim**	**Methods**	**Current status**
Lymphatic system health in Alzheimer's disease (ID: NCT04205539)	To characterize differences in lymphatic health among Alzheimer's disease patients	Using dexmedetomidine, a short-acting agent, to induce a sleep-like state to assess interstitial fluid convection and free water diffusion in patients	Withdrawn due to the COVID-19 pandemic, no new updates posted since 2022
Quantitative imaging of brain glymphatic function in humans (ID: NCT04768101)	To assess the relationship between Alzheimer's disease related dementias and CNS lymphatic function in patients	A novel magnetic resonance imaging method that is sensitive to lymphatic drainage dysfunction will be used	Currently recruiting for this Phase I study that intends to reach completion by late 2024
“Effects of exercise on glymphatic functioning and neurobehavioral correlates in Parkinson's disease (FIGHTPD)” (ID: NCT04140708)	To test if exercise potentiates glymphatic function and would therefore increase amyloid beta clearance from the brain and improve neurobehavioral outcomes	Participants with Parkinson's disease are invited to attend a weekly boxing class for 12 weeks, following which their beta-amyloid levels will be measured via MRI/PET scans and compared to baseline measurements	The first phase of this study is expected to reach completion by late summer 2023
The role of meningeal lymphatic vessels in the absorption of chronic subdural hematoma and its injury mechanism (ID: NCT05426889)	To discover the role that lymphatics play in the absorption of chronic subdural hematomas and use the knowledge to develop targeted treatment down the line	Patients who chose to undergo removal of large hematomas will undergo measurement of the signal intensity of the meningeal lymphatic vessels for up to 6 months post-surgery	This study is actively recruiting participants
Meningeal inflammation on 7T MRI as a tool for measuring and predicting Ocrelizumab response in Multiple Sclerosis (ID: NCT03396822)	To identify meningeal inflammation as a predictor for the effectiveness of the drug ocrelizumab for treatment of Multiple Sclerosis.	To use 7 tesla MRI to visualize if the use of ocrelizumab, which destroys B-cells, may reduce meningeal inflammation levels in patients	This Phase I study is currently active but not recruiting; it was expected to reach completion by March 2023, but an update has not yet been posted

## Perspectives

The understanding of CNS immunity has come a long way from the conception of the CNS as a strictly “immune privileged” organ. As the brain does not contain lymphatic vessels, scientists believed that antigens could be introduced into the system without inducing an inflammatory immune response, giving the impression that the brain alone cannot potentiate a robust immune response. New and emerging knowledge about the meningeal lymphatic system has highlighted and characterized the movement of immune cells in this system in both a healthy and diseased brain environment. Understanding the unique features of this system will not only permit a greater understanding of its functions but could also provide avenues for developing novel therapeutics for CNS disorders. A current research study on CNS disorders suggests that aberrations in lymphatic drainage may contribute to the symptoms of certain neurological diseases including Alzheimer's disease, Parkinson's disease, TBI, and brain tumors. The manipulation of lymphangiogenesis could be a target for treatment, perhaps through increasing the formation of lymphatic vessels to aid in the clearance of harmful debris from the lymphatic system. Moreover, the parallel rediscovery and recharacterization of the gut microbiome in the past decade, especially in the unraveling of the microbiota's important roles in the development and function of the immune system, brings up new questions on a potential gut-meningeal immunity communication wherein the gut microbiota may play a role in shaping the immune populations present in the meninges. Research in this field is already underway, with one key study published in *Nature* in 2020, demonstrating how gut-educated IgA cells traveled through the bloodstream to reach distant tissues and organs, including the meninges of the brain, to produce local antibodies (Fitzpatrick et al., 2020). This bears remarkable significance to meningeal immunity, suggesting that a route for the modulation of meningeal lymphatics may originate within the gut, wherein the microbiota could be therapeutically targeted to modulate immune changes that would ultimately protect the brain. In conclusion, this field of knowledge is rapidly expanding as is the potential for improved and more effective treatments leveraging meningeal lymphatic modulations that may catalyze a major breakthrough in the clinic.

## Author contributions

AM and LL: design, literature review and interpretation, manuscript writing, and the final approval of the manuscript. VK and RA: manuscript writing and final approval of the manuscript. KS: conception and design, literature review and interpretation, manuscript writing, and the final approval of the manuscript. All authors contributed to the article and approved the submitted version.
